# Structural brain abnormalities in schizophrenia patients with a history and presence of auditory verbal hallucination

**DOI:** 10.1038/s41398-022-02282-5

**Published:** 2022-12-22

**Authors:** Mari Sone, Daisuke Koshiyama, Yinghan Zhu, Norihide Maikusa, Naohiro Okada, Osamu Abe, Hidenori Yamasue, Kiyoto Kasai, Shinsuke Koike

**Affiliations:** 1 Center for Evolutionary Cognitive Sciences, Graduate School of Art and Sciences, The University of Tokyo, Meguro-ku, Tokyo, 153-8902 Japan; 2 Department of Neuropsychiatry, Graduate School of Medicine, The University of Tokyo, Bunkyo-ku, Tokyo, 113-8655 Japan; 3 The International Research Center for Neurointelligence (WPI-IRCN), Institutes for Advanced Study (UTIAS), The University of Tokyo, Bunkyo-ku, Tokyo, 113-0033 Japan; 4 Department of Radiology, Graduate School of Medicine, The University of Tokyo, Bunkyo-ku, Tokyo, 113-8655 Japan; 5 Department of Psychiatry, Hamamatsu University School of Medicine, Hamamatsu City, 431-3192 Japan; 6 University of Tokyo Institute for Diversity & Adaptation of Human Mind (UTIDAHM), Meguro-ku, Tokyo, 153-8902 Japan; 7 University of Tokyo Center for Integrative Science of Human Behavior (CiSHuB), Meguro-ku, Tokyo, 153-8902 Japan

**Keywords:** Molecular neuroscience, Schizophrenia

## Abstract

Although many studies have demonstrated structural brain abnormalities associated with auditory verbal hallucinations (AVH) in schizophrenia, the results remain inconsistent because of the small sample sizes and the reliability of clinical interviews. We compared brain morphometries in 204 participants, including 58 schizophrenia patients with a history of AVH (AVH + ), 29 without a history of AVH (AVH−), and 117 healthy controls (HCs) based on a detailed inspection of medical records. We further divided the AVH+ group into 37 patients with and 21 patients without hallucinations at the time of the MRI scans (AVH++ and AVH+−, respectively) via clinical interviews to explore the morphological differences according to the persistence of AVH. The AVH + group had a smaller surface area in the left caudal middle frontal gyrus (*F* = 7.28, *FDR-corrected p* = 0.0008) and precentral gyrus (*F* = 7.68, *FDR-corrected p* = 0.0006) compared to the AVH− group. The AVH+ patients had a smaller surface area in the left insula (*F* = 7.06, *FDR-corrected p* = 0.001) and a smaller subcortical volume in the bilateral hippocampus (right: *F* = 13.34, *FDR-corrected p* = 0.00003; left: *F* = 6.80, *FDR-corrected p* = 0.001) compared to the HC group. Of these significantly altered areas, the AVH++ group showed significantly smaller bilateral hippocampal volumes compared to the AVH+− group, and a smaller surface area in the left precentral gyrus and caudal middle frontal gyrus compared to the AVH- group. Our findings highlighted the distinct pattern of structural alteration between the history and presence of AVH in schizophrenia, and the importance of integrating multiple criteria to elucidate the neuroanatomical mechanisms.

## Introduction

Auditory verbal hallucination (AVH) is a cardinal feature of schizophrenia; in AVH, patients hear voices without the presence of corresponding external stimuli [1]. A number of magnetic resonance imaging (MRI) studies have compared the brain morphology of schizophrenia patients with and without AVH, and their findings suggest the presence of structural and functional alterations in regions such as the superior temporal gyrus, hippocampus, insula, anterior cingulate, inferior frontal gyrus, and inferior temporal gyrus in those with AVH [2–5].

One of the most consistent findings in AVH is the involvement of language-related regions such as the speech production and perception areas [6]. For example, a meta-analysis of AVH fMRI studies showed that AVH was associated with increased activities in speech generation areas such as the inferior frontal gyrus and insula [7]. Another language-related region that has elicited “renewed interest” in recent years is the middle frontal gyrus (MFG), particularly the posterior MFG and Broadman area (BA) 55b [8]. BA55b comprises the caudal part of MFG and precentral gyrus, and it actively participates in word production and verbal working memory [8–10]. Although the structural and functional relationship of MFG with AVH has been confirmed [11, 12]. its role in AVH has not been sufficiently discussed.

In addition to speech generation, one of the most influential hypotheses suggests the importance of speech perception in AVH [13, 14]. According to the theory, AVH is caused by the inability of patients to distinguish self-produced voices from external voices [15]. In support of this hypothesis, some experiments have demonstrated this impaired ability in perception among those with AVH. For example, Brébion et al. reported that patients with schizophrenia were prone to confuse their own verbal production with verbal production by experimenters or even pictorial representations [16]. Moreover, the same experiment demonstrated a correlation between hallucination severity and false recognition rate [16]. Morphological abnormalities in the speech perception regions, such as the medial prefrontal cortex, medial parietal cortex, middle temporal gyrus (MTG), and superior temporal gyrus, have also been confirmed in AVH individuals [14, 17, 18].

Another theory suggests the alteration in the default mode network as a trigger of AVH. The default mode network is a well-known resting network that is active when people are engaged in the internally directed tasks such as autobiographical memory recollection [19]. The network consists of regions such as the dorsal medial prefrontal cortex, posterior cingulate cortex, precuneus, inferior parietal lobe, lateral temporal cortex, and hippocampus [20], and evidences have shown its disintegration during AVH [21]. For example, a study demonstrated the withdrawal of the default mode network during AVH and showed that the instability of the default mode network and the severity of hallucination were correlated [22]. AVH-related dysfunction of the default mode network has been implicated as a possible hypothesis regarding AVH [23].

Although substantial effort has been made in AVH studies, results have been inconsistent. While previous studies often report morphological alterations in regions including the superior temporal gyrus, insula, anterior cingulate, and hippocampus for people who experience AVH [2–5], many studies have not confirmed these findings [24–26]. These inconsistent findings in morphometric studies may be due to small sample sizes (*n* < 20) [27–29] and differences in the definition of the AVH group. While some researchers adopt AVH group criteria as the presence of an AVH history [30, 31], others consider the AVH group as the presence of AVH at the time of scanning [6, 32]. As AVH symptoms are treatable, there might be some discrepancy between the two criteria. Previous studies have adopted either criteria; thus, analysis including both criteria might shed new light on aspects of AVH. Moreover, studies that assessed the presence of AVH history used only clinical interviews during the MRI scan, which may have introduced recall bias. Therefore, more reliable assessments, such as detailed medical records at the first visit and on admission, are needed. In this study, we utilized detailed examination of medical records and clinical interviews to assess the presence of AVH in order to diminish potential biases.

This study aimed to elucidate AVH-specific structural alterations in schizophrenia patients with a history of AVH (AVH + ), patients without AVH (AVH-), and healthy controls (HCs). To evaluate the presence of AVH history, we obtained detailed illness history from their medical records, including records at the first visit and clinical follow-up visits in the outpatient unit, and at admissions and discharges in the inpatient unit. We further divided the AVH+ group into those with or without hallucinations at the time of the MRI scan (AVH++ and AVH+−, respectively) to explore potential morphological discrepancies according to the persistence of AVH. We hypothesized that regions relevant to speech production and perception would be structurally altered in AVH+ individuals. More specifically, we expected that the cortical thickness and surface area in the regions such as the inferior frontal gyrus, insula, MFG, medial prefrontal cortex, medial parietal cortex, MTG, and superior temporal gyrus would be smaller in AVH+ than AVH−. Furthermore, we expected that these regions would be smaller in patients with AVH++ than AVH+−.

## Materials and methods

### Participants

The study comprised 204 participants, including 87 individuals with schizophrenia and 117 HCs matched for sex, age, intelligence quotient (IQ), and handedness (*p* > 0.10, Table 1). All patients were recruited from the University of Tokyo Hospital. Psychiatric diagnoses were made based on the Diagnostic and Statistical Manual of Mental Disorders IV TR [33] by experienced psychiatrists. We then divided the 87 schizophrenia patients based on their history of AVH into those with (*n* = 58, AVH+ ) and without (*n* = 29, AVH−) a history of AVH. Furthermore, those with AVH were divided into individuals with (*n* = 37, AVH++ ) and without (*n* = 21, AVH+−) the presence of hallucinations at the time of the MRI scan.

**Table 1:** Demographic and clinical characteristics of the study participants.

Subgroups	Healthy controls		Schizophrenia								Statistics for 3 groups		Statistics for 4 subgroups	
			AVH−		AVH+		AVH+−		AVH++					
					total									
	Mean	SD	Mean	SD	Mean	SD	Mean	SD	Mean	SD	Statistical value	*p*-value	Statistical value	*p*-value
*n*	117		29		58		21		37					
Male/Female	64/53		20/9		31/27		14/7		17/20		*χ*^2^ = 2.2	0.33	*χ*^2^ = 4.5	0.20
Age (years)	29.5	8.2	28.2	9.1	31.8	9.6	30.1	9.9	32.7	9.4	*F* = 1.96	0.14	*F* = 1.69	0.17
Handedness: right/mixed/left	97/17/ 3		26/1/2		48/9/1		18/0/3		30/1/6		*χ*^2^ = 4.5	0.34	*χ*^2^ = 4.9	0.56
JART25 IQ	104.5	7	102.1	8.8	102.2	9.6	100.3	9.2	103.2	9.7	*F* = 2.1	0.13	*F* = 2.0	0.12
Chlorpromazine eq. dose (mg)	NA	NA	593.8	493.6	699.1	614.4	377.6	251.3	881.6	684.2	*t* = 0.86	0.39	*F* = 5.6	0.005*
Duration of illness (years)	NA	NA	4.2	7.2	6.6	6.4	5.24	5.45	7.38	6.83	*t* = 1.5	0.14	*F* = 2.1	0.13
PANSS														
Positive symptom	NA	NA	15.7	4.8	17.1	5.2	12.6	4.0	18.8	5.9	*t* = 1.3	0.2	*F* = 8.81	< 0.001*
Negative symptom	NA	NA	20.5	5.8	20.8	6.6	18.4	8.9	21.6	5.7	*t* = 0.23	0.82	*F* = 0.84	0.44
General Psychopathology	NA	NA	36.3	9.5	37.3	9.4	31.5	11.9	39.5	8.5	*t* = 0.47	0.64	*F* = 3.4	0.040*

*PANSS* Positive and Negative Syndrome Scale, *JART25 IQ* Estimated (premorbid) intelligent quotient assessed using the 25-item version of the Japanese Adult Reading Test, *AVH* + Schizophrenia patients with a history of auditory verbal hallucination, *AVH*− Schizophrenia patients without a history of auditory verbal hallucination.

The history of AVH was assessed based on a clear description of the presence of AVH in the medical records. Detailed illness histories of the participants were obtained by experienced psychiatrists during the patients’ first visits to the outpatient unit of the University of Tokyo Hospital, and from all admissions and discharges in the inpatient unit. All clinical follow-up records were also examined. The existence of AVH was evaluated by two experienced psychiatrists (D.K. and S.K.) using the medical records. The presence of AVH was defined as a clear description of the existence of AVH in their medical records. The reproducibility of the evaluation using a random sample of 50 patients was high (kappa = 0.88), and all disagreements and/or unclear categorizations were discussed and concluded by the two psychiatrists. We further examined the medical records one month before and after their scans. The records in the AVH++ individuals were more likely to be stated about AVH presence compared to those in the AVH+− individuals (*χ*^*2*^ = 3.87, *df* = 1, *p* = 0.049).

Psychiatric symptoms were assessed by experienced psychiatrists or psychologists using the Positive and Negative Syndrome Scale (PANSS) a week before the MRI scan. The presence of hallucinatory behavior at the time of MRI was defined according to the PANSS P3 score, and a score of ≤ 2 indicated AVH+− and while ≥ 3 indicated AVH++.

The exclusion criteria were as follows: history of neurological disease, illegal drug or alcohol abuse, head trauma accompanied by loss of consciousness, or mass anomalies and/or abnormalities noted on conventional diagnostic MRI. Trained psychiatrists or psychologists screened all HC participants using the Japanese version of the Mini International Neuropsychiatric Interview [34, 35] to exclude those who had any psychiatric disorders. For all participants, handedness was assessed using the rating scale of handedness or the Edinburgh Inventory [36], and their scores were transformed into +100 (right-handed) to −100 (left). The (premorbid) estimated IQ was assessed for all participants with the 25-item version of the Japanese Adult Reading Test (JART) [37]. Medication doses in the schizophrenia groups were calculated using the chlorpromazine equivalent dose [38].

This study was approved by the Ethics Committee of Graduate School of Medicine, The University of Tokyo (No. 397, 2226, and 3150). Written informed consent was obtained from all participants before participation of the study.

### Image acquisition

All structural MR images were acquired by two procedures using 3 Tesla GE scanners (GE Healthcare, Chicago, Il) and a T1-weighted 3D-fast spoiled gradient echo sequence. For protocol 1, we examined 106 participants (HC = 58, AVH+ = 30, AVH− = 18) and used the following equipment and parameters: SIGNA HDx with an 8-channel head coil, TR = 6.80 ms, TE = 1.94 ms, flip angle = 20°, slice thickness = 1.0 mm, FOV = 240 × 240 mm, and number of slices = 176. For protocol 2 (total = 98, HC = 59, AVH+ = 28, AVH− = 11), we used DISCOVERY MR750w with a 24-channel head coil, TR = 7.65 ms, TE = 3.10 ms, flip angle = 11°; slice thickness = 1.2 mm, FOV = 256 × 256 mm, and number of slices = 196. There were no procedural differences in the number of groups (*χ*^2^ = 1.5, p = 0.5).

### MRI data processing

For quality control purposes, a visual examination was conducted to detect any abnormality. After data processing using FreeSurfer version 6.0.0 [39], we examined 150 neuroimaging measures as follows: 68 cortical thickness, 68 surface area, and 14 subcortical volume according to the Desikan-Killiany atlas [40]. To correct the measurement bias caused by scanner or image protocol difference, we used a ComBat harmonization method for the two datasets [41].

### Statistical analysis

All statistical analyses were conducted using R version 4.0.1. We examined group differences based on AVH history using an analysis of variance (ANOVA) with each MRI measure (i.e., cortical thickness, surface area, and subcortical volume) as the dependent variable, group (AVH+ , AVH−, and HC) as the independent variable, and estimated intracranial volume as a covariate. Multiple comparisons (*n* = 150) were corrected using the false discovery rate (FDR). We then conducted post hoc analyses for the structural brain measures that showed significant group differences using the Tukey honestly significant difference (HSD) test. We further tested for significant differences in brain measures between the three clinical subgroups (AVH−, AVH++ , and AVH+−) using one-way ANOVAs and FDR correction. Moreover, we computed laterality index, an index of hemispheric dominance, to test whether the laterality of structural brain measures would differ between the existence of AVH. We computed the index in the significant regions of interest as follows:

$${\it{Laterality}}\,{\it{Index}} = \left( {L - R} \right)/(L + R),$$

One-way ANOVAs were then conducted to see the potential significant difference in the laterality indices between the three groups (AVH−, AVH+ , and HC).

For significant differences in brain measures, a general linear model was used to ascertain the common and group-specific relationship between brain measures and demographic and clinical variables such as age, sex, the PANSS sub-scores (positive, negative, general psychopathology, and P3), duration of illness, and chlorpromazine equivalent dose. In the models, we included main effect of group (AVH−, AVH+ , and HC [if applicable]) and each demographic (or clinical) variable, and demographic variable by group interaction as the independent variables, and estimated intracranial volume as a covariate, and applied FDR correction.

## Results

### Demographic characteristics

Sex, age, estimated IQ, and handedness did not differ between the groups (Table 1). The AVH + and AVH- gSsroups did not differ in chlorpromazine equivalent doses, duration of illness, and PANSS sub-scores (*p’s* > 0.05). For the three clinical subgroups (AVH−, AVH+−, and AVH++ ), the AVH++ group had significantly greater PANSS positive score compared to the AVH+− and AVH− groups (*F* = 12.8, *p* < 0.001; *post-hoc Tukey HSD adjusted p* < 0.001 and 0.0048, respectively) and more chlorpromazine equivalent dose compared to the AVH+− group (*F* = 5.6, *p* = 0.0052; *post-hoc Tukey HSD adjusted p* = 0.0053).

### Difference in brain measures

**Fig. 1:**
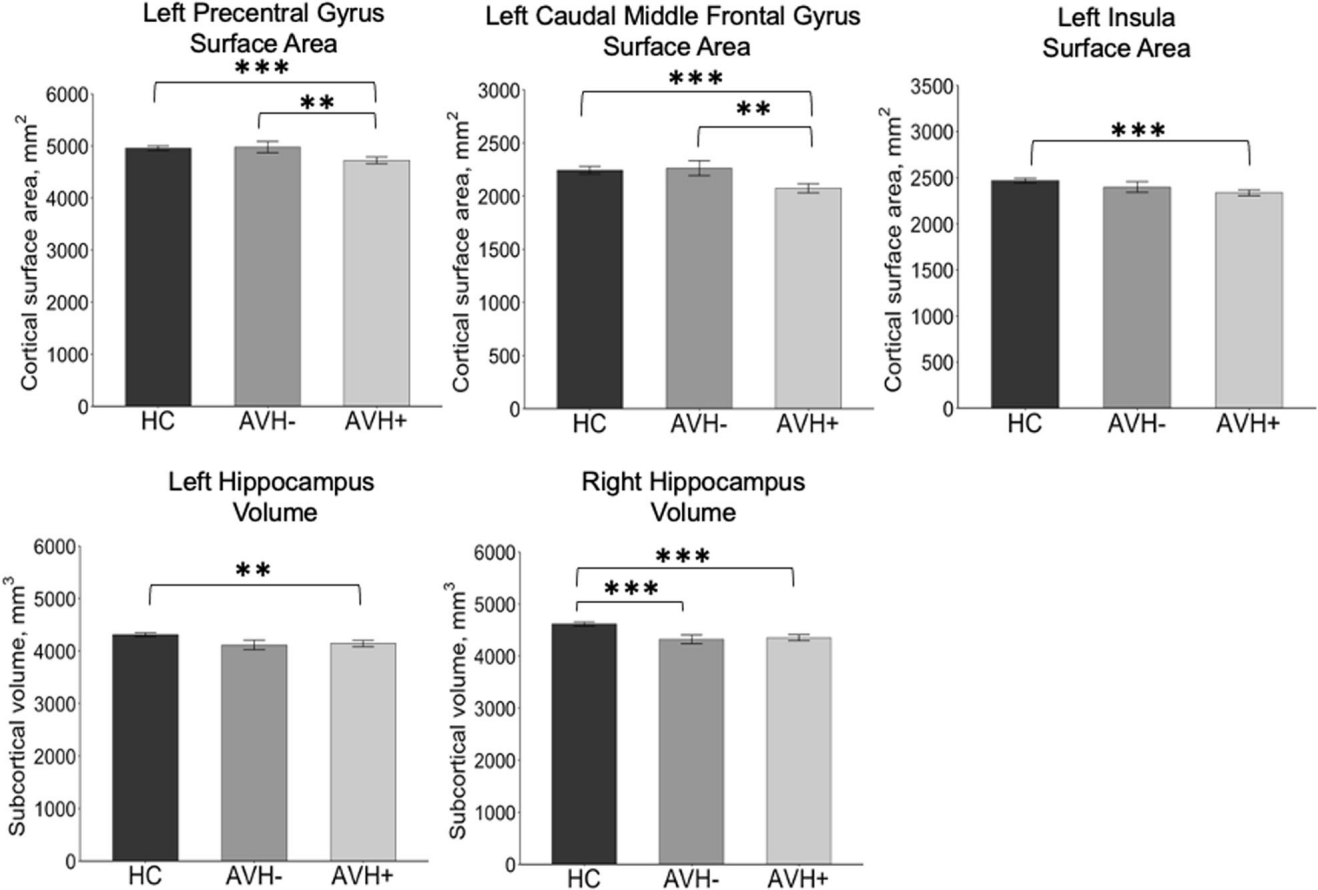
Morphometric differences between the groups. *p < 0.05, **p < 0.01, ***p < 0.001. HC Healthy control, AVH + Schizophrenia patients with a history of auditory verbal hallucinations, AVH − Schizophrenia patients without a history of auditory verbal hallucinations.

ANOVA showed significant group differences in the surface area of the left precentral gyrus (*F* = 7.68, *FDR-corrected p* = 0.0006) and left caudal MFG (*F* = 7.28, *FDR-corrected p* = 0.0008). In these regions, the AVH+ group had a significantly smaller area than the AVH− group (left precentral gyrus surface area: *post-hoc Tukey HSD adjusted p* = 0.01, left caudal MFG surface area: *post-hoc Tukey HSD adjusted p* = 0.01) and HC group (left precentral gyrus surface area: *post-hoc Tukey HSD adjusted p* = 0.0008, caudal MFG surface area: *post-hoc Tukey HSD adjusted p* = 0.001). Significantly smaller brain measures in the AVH + group compared to the HC group were also found for right hippocampal volume (*F* = 13.34, *FDR-corrected p* = 0.000003; *post-hoc Tukey HSD adjusted p* = 0.00006; Fig. 1), left hippocampal volume (*F* = 6.80, *FDR-corrected p* = 0.001; *post-hoc Tukey HSD adjusted p* = 0.007), and left insular surface area (*F* = 7.06, *FDR-corrected p* = 0.001; *post-hoc Tukey HSD adjusted p* = 0.0008) but not AVH + compared to the AVH- group (*uncorrected p* > 0.05). No significant differences were found regarding the cortical thickness measures (*uncorrected p* > 0.05).

**Fig. 2:**
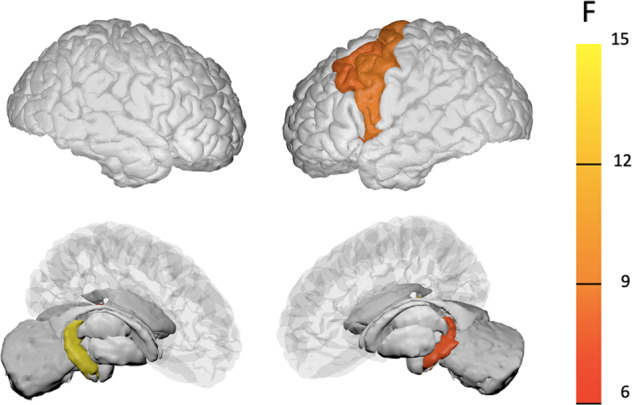
Brain regions with significant morphometric differences between the groups.

One-way ANOVA and the following post-hoc analysis for the five brain measures showed that the AVH++ group had significantly smaller subcortical volumes in the bilateral hippocampus than the AVH+− group (right: *F* = 4.26, *FDR-corrected p* = 0.017, *post-hoc Tukey HSD adjusted p* = 0.01; left: *F* = 4.95, *FDR-corrected p* = 0.009, *post-hoc Tukey HSD adjusted p* = 0.007). The surface areas in the left precentral gyrus (*F* = 4.02, *FDR-corrected p* = 0.021; *post-hoc Tukey HSD adjusted p* = 0.02) and left caudal MFG (*F* = 4.24, *FDR-corrected p* = 0.018; *post-hoc Tukey HSD adjusted p* = 0.02) were significantly smaller in the AVH++ group than in the AVH − group (Fig. 2). The surface area in the left insula did not differ between the groups (*F* = 0.77, *p* = 0.46). After controlling for chlorpromazine equivalent dose, the differences still survived the FDR correction in the four measures (*FDR-corrected p* < 0.05). We computed the laterality indices for the four significant regions, and one-way ANOVAs showed no significant differences in any regions (*FDR-corrected p* > 0.05) but a trend-level difference in the caudal MFG surface area (*uncorrected p* = 0.048). A post-hoc Tukey HSD test also showed a trend-level difference between AVH+ and HC groups (*adjusted p* = 0.062).

### The relationship between brain measures and demographic and clinical variables

For the five significant brain regions (*q* < 0.05, *p* < 0.01 = 0.05/5), general linear models for age and the 3 groups (HC, AVH− , and AVH+ ) showed no main effect of age or age by group interaction in any regions. General linear models showed significant main effect of sex in the surface area of the left precentral gyrus (*t* = −2.81, *FDR-corrected* *p* = 0.005), and right hippocampus (*t* = −3.98, *FDR-corrected* *p* < 0.0001) but not sex by group interaction. General linear models for duration illnesses and the 2 groups (AVH− and AVH+ ) showed a main effect of the duration of illnesses in the surface area of the left caudal MFG (*t* = −3.13, *FDR-corrected p* = 0.002) but not the duration of illnesses by group interaction. General linear models for the PANSS negative symptom subscale showed the main effect of the score in the surface area of the left MFG (*t* = −2.64, *FDR-corrected* *p* = 0.0099) and right hippocampus (*t* = −2.86, *FDR-corrected* *p* = 0.005). In addition, the interaction of score by group was found in the surface area of left caudal MFG (*t* = 2.93, *corrected p* = 0.004). For each group, there was no significant correlation between the PANSS negative score and the surface area but the opposite coefficient trend (AVH-: *r* = −0.25, *p* = 0.19; AVH+ : r = 0.13, *p* = 0.32). For chlorpromazine equivalent dose, and the other PANSS sub-scales (positive, general, and P3), there was no main effect of the clinical variables or interaction in any regions.

## Discussion

The current study investigated the specific alterations in schizophrenia patients with a history and/or presence of AVH. We found that the surface areas in the left precentral gyrus and left caudal MFG were significantly smaller in AVH + patients than in the AVH− and HC groups. The surface area in the left precentral gyrus and the volume in the bilateral hippocampus were also significantly smaller in the AVH+ group than in the HC group. Further analysis found that AVH++ individuals had smaller bilateral hippocampal volume compared to the AVH+− group, and a smaller surface area in the left precentral gyrus and caudal MFG compared to the AVH− group.

To the best of our knowledge, this is the first study to investigate structural abnormalities related to AVH based on a detailed assessment of medical records and standardized clinical interviews. Since previous studies on AVH in schizophrenia employed only clinical interviews at the time of the MRI scans, especially concerning a history of AVH, recall bias may have affected the validity of their findings. Furthermore, in addition to the structural abnormalities based on AVH history, structural alterations depending on the presence of AVH at the time of the MRI scan shed light on the importance of integrating the two criteria to elucidate neurological alterations and potential mechanisms of AVH.

Our results suggested that the left precentral gyrus and left caudal MFG were specifically altered in AVH. Indeed, structural or functional abnormalities in these regions in AVH patients have been confirmed in previous studies [11, 12]. The regions include BA55b, which is known for its active involvement in language manipulation [8, 10], especially regarding speech production [42]. Furthermore, the caudal MFG is known to be associated with verbal working memory [43]. Another prominent finding regarding the left MFG is its role in verbal cognitive processing [44]. In addition to its role in the “cortical organization” for language [45], the caudal MFG is associated with cognitive selection among competing stimuli, including both visual and auditory stimuli [17, 18]. It has been reported as a “key prefrontal area”, accounting for higher-order selection between stimuli [17]. For example, the thickness of the area was associated with the performance of dichotic listening, with cortical thinning negatively affecting this ability [46]. Interestingly, structural abnormalities of the left MFG are also associated with unawareness of illness, making it difficult for patients with schizophrenia to recognize their mental illness or reality [47, 48].

AVH is considered to be caused by a lack of self-monitoring ability, with patients misattributing their own verbal thoughts as voices from external source(s) [13, 14]. Indeed, experiments have shown that patients with AVH had an impaired ability to differentiate speech they produced by themselves from speech produced by others [16]. Considering the previously discussed characteristics of BA55b and caudal MFG, structural abnormality in this region may contribute to the deterioration of the ability to correctly attribute self-produced words and external stimuli.

Another potential mechanism is the contribution of the caudal MFG to the default mode network. One of the popular theories on AVH suggests an aberrant default mode network in AVH generation. This theory was established because of the increasing evidence on the altered resting-state connectivity demonstrated by individuals with AVH [21]. For example, Jardri et al. reported that spontaneous withdrawal of the default mode network during the AVH experience and the instabilities of the network were correlated with hallucination severity [22]. Other studies also confirm a similar phenomenon, where the relevance of the default mode network is becoming one of the convincing models [23]. Repetitive transcranial magnetic stimulation on the temporo-parietal junction is one of the effective treatments for AVH amelioration [49]. Interestingly, Bais et al reported that repetitive left-sided transcranial magnetic stimulation allowed the left MFG to contribute to a stronger default mode network [50]. The result highlights the potential role of the left MFG in the augmentation of default mode network connectivity. Another study also confirmed decreased connectivity of the MFG in the default mode network for the AVH+ group in comparison with the AVH− group [51]. Considering these results, we postulate that the morphological abnormality in the left MFG might contribute to the abnormal connectivity of the default mode network, which may significantly affect AVH generation.

Our results which showed hippocampal morphometric difference with AVH persistence further support the crucial involvement of the hippocampus in AVH production. However, the AVH++ group showed severer positive symptoms and general psychopathology, and greater medication dose than the AVH+− group. Thus, the effect of symptom severity and treatment resistance trait on the volume differences should be considered. The relationship between morphological abnormality in the hippocampus and symptom severity [52] and treatment resistance trait [53, 54] have been reported. In this study, there was no relationship between hippocampal volumes and clinical severity in any group or overall patients with schizophrenia. Therefore, the difference between AVH++ and AVH+− may suggest an AVH persistence-specific feature, but we need to test the potential effect of AVH persistence, symptom severity, and treatment resistance on the hippocampus volumes in the future investigations.

Another influential hypothesis suggests that dysfunction in the hippocampus might drive dopamine dysregulation in psychotic symptoms [55]. Indeed, the association of AVH and abnormalities in dopamine release is well documented [56, 57]. A study confirmed that dopamine augmentation induced hallucination-like behavior in mice [58]. A finding showed that reduction in hippocampal parvalbumin expression induced a strengthened dopamine system in the ventral tegmental area and positive symptom-like behavior in rats [59]. The thalamo-hippocampal-ventral tegmental area loop may be an underlying circuit for the onset of psychotic symptoms [60]. As the ventral tegmental area is a key region for dopamine function which often plays a role in psychiatric disorders [61], it is possible that dysfunction in the hippocampus can trigger the abnormal function of dopaminergic systems, which may induce AVH generation.

Our study has some limitations. First, our criteria for the presence/absence of hallucinations at the time of MRI scan was derived from the PANSS P3 score which measures the extent of hallucinatory behavior, including visual hallucinations. As the criteria for AVH history based on medical records excluded other types of hallucinations, the two criteria may not fully correspond. Second, clinical data were collected from multiple scanners. Although we previously confirmed that the ComBat approach diminishes the measurement bias to a small effect size compared to the difference between test-retest scans from the same individuals [41], the presence of potential measurement and sampling biases cannot be excluded. Third, many participants in this study were on medication. Although the analysis showed no significant between-group differences in medication intake or a correlation between the medication and structural measures, it would be informative to conduct future studies that clarify the effect of medication on the structural alteration in schizophrenia for clearer conclusions.

In conclusion, this study is the first to investigate structural alterations in AVH according to the history and presence of AVH obtained from the combination of medical record examinations and clinical interviews conducted at the time of MRI scan. Patients with a history of AVH showed distinct structural abnormalities compared to the non-AVH and HC group in the left caudal MFG, left precentral cortex, left insula, and bilateral hippocampus. Moreover, a distinct pattern of structural alteration was noted depending on the persistence of AVH. Our findings provide new insights into structural alterations in AVH and highlight the importance of integrating multiple criteria to elucidate the neuroanatomical mechanisms of AVH in future studies.
